# DS1/OsEMF1 interacts with OsARF11 to control rice architecture by regulation of brassinosteroid signaling

**DOI:** 10.1186/s12284-018-0239-9

**Published:** 2018-08-06

**Authors:** X. Liu, C. Y. Yang, R. Miao, C. L. Zhou, P. H. Cao, J. Lan, X. J. Zhu, C. L. Mou, Y. S. Huang, S. J. Liu, Y. L. Tian, T. L. Nguyen, L. Jiang, J. M. Wan

**Affiliations:** 1 State Key Laboratory for Crop Genetics and Germplasm Enhancement, Jiangsu Plant Gene Engineering Research Center, Nanjing Agricultural University, Nanjing, 210095 China; 2 National Key Facility for Crop Gene Resources and Genetic Improvement, Institute of Crop Science, Chinese Academy of Agricultural Sciences, Beijing, 100081 China

**Keywords:** Brassinosteroids (BRs), Plant architecture, *Oryza sativa*, *D61*/*OsBRI1*

## Abstract

**Background:**

Plant height and leaf angle are important determinants of yield in rice (*Oryza sativa* L.). Genes involved in regulating plant height and leaf angle were identified in previous studies; however, there are many remaining unknown factors that affect rice architecture.

**Results:**

In this study, we characterized a dwarf mutant named *ds1* with small grain size and decreased leaf angle,selected from an irradiated population of ssp. *japonica* variety Nanjing35. The *ds1* mutant also showed abnormal floral organs. *ds1* plants were insensitive to BL treatment and expression of genes related to BR signaling was changed. An F_2_ population from a cross between *ds1* and *indica* cultivar 93–11 was used to fine map *DS1* and to map-based clone the *DS1* allele, which encoded an EMF1-like protein that acted as a transcriptional regulator. *DS1* was constitutively expressed in various tissues, and especially highly expressed in young leaves, panicles and seeds. We showed that the DS1 protein interacted with auxin response factor 11 (OsARF11), a major transcriptional regulator of plant height and leaf angle, to co-regulate *D61*/*OsBRI1* expression. These findings provide novel insights into understanding the molecular mechanisms by which *DS1* integrates auxin and brassinosteroid signaling in rice.

**Conclusion:**

The *DS1* gene encoded an EMF1-like protein in rice. The *ds1* mutation altered the expression of genes related to BR signaling, and *ds1 *was insensitive to BL treatment. DS1 interacts with OsARF11 to co-regulate *OsBRI1* expression.

**Electronic supplementary material:**

The online version of this article (10.1186/s12284-018-0239-9) contains supplementary material, which is available to authorized users.

## Background

Rice is an important food crop that feeds more than half of the world population. With an increasing global population, food security and safety are becoming serious issues. Given the limited availability of arable land and the growing human population, a future increase in rice production will be a major challenge for rice breeders (Wang et al. 2008). Plant height and leaf angle are important agronomic traits directly affecting grain yield and crop architecture, which are major objectives of crop improvement (Ikeda et al. 2013).

Plant height, an important trait in crop improvement, is related to lodging resistance, grain yield, and biomass production. Reduced plant height and associated lodging resistance are predominant strategies in crop improvement (Ayano et al. 2014). Various factors cause reduced height in plants; gibberellin (GA) and brassinosteroids (BRs) are the most widely investigated factors affecting plant height in rice. Many rice GA- and BR-related mutants show dwarf or semi-dwarf phenotypes, such as *sd1*, *d1* and *d61* (Sasaki et al. 2002; Fujisawa et al. 1999; Zhang et al. 2016; Yamamuro et al. 2000). These dwarf mutants result from reduced cell numbers or cell length in stems, and analyzing more dwarf mutants may provide novel insights into the mechanisms controlling stem elongation.

Leaf angle, the degree of bending between the leaf blade and culm, is a key factor determining the plant architecture and grain yield (Zhao et al. 2010). Crops with erect leaves have increased photosynthetic effciency and nitrogen storage for grain flling and are suitable for dense planting (Sakamoto et al. 2006). Many genes or QTLs have been reported to control leaf angle, including *D61*/*OsBRI1*, *ILI1*, *LC2*, *ILA1*, *RAV6*, *OsARF19*, and *SLG* (Yamamuro et al. 2000; Zhang et al. 2009; Zhao et al. 2010; Ning et al. 2011; Zhang et al. 2015b; Zhang et al. 2015a; Feng et al. 2016). Most identified rice mutants with altered leaf inclination have abnormal cell division and or expansion and altered cell wall composition at the leaf:stem joint (Zhang et al. 2009; Zhao et al. 2010). Nevertheless, BR and auxin synergistically control leaf inclination in rice (Wada et al. 1981; Hirano et al. 2017).

The synergetic effects of auxin and BRs on various physiological events have strongly suggested the interdependency of their signaling, and molecular evidence for this has been reported. For example, the auxin-inducible genes, *indole-5-acetic acid* (*IAA5)*, *IAA19* and *SAUR-AC1*, are induced by brassinolide (BL) treatment, whereas the expression of *IAA5* and *IAA19* are down-regulated in the *Arabidopsis* BR-deficient mutant *de-etiolated 2* (*det2*) (Nakamura et al. 2003). In addition, expression of BR biosynthetic gene *DWARF4* and the BR receptor *BRI1* are induced by auxin (Park et al. 2011; Sakamoto et al. 2012). *BRASSINAZOLE RESISTANT 1* (*BZR1*) directly regulates many auxin-responsive genes (Sun et al. 2010). Nemhauser et al. (2004) found that 48 genes were co-regulated by auxin and BL (e.g. *SAUR*, *Aux/IAA* and *GH3*). They also found that a TGTCTC element in the auxin-responsive element (AuxRE) was enriched in genes up-regulated by both IAA and BL. These results strongly suggest that the two hormone pathways synergistically affect gene expression at the transcriptional level.

In this study, we identified a reduced BR-sensitivity mutant, *ds1*, showing dwarfness, smaller seed and decreased leaf angle compared to wild type. The small plant stature of the plants and decreased leaf angle resulted from reduced cell elongation. Our genetic mapping and molecular biology experiments revealed that the underlying *DS1*, a new allele of *OsEMF1*, encodes an EMF1-like protein involved in the development of plant height and leaf angle by interacting with OsARF11 to co-regulate *OsBRI1* expression.

## Results

### Characterization of the *ds1* mutant

**Fig. 1:**
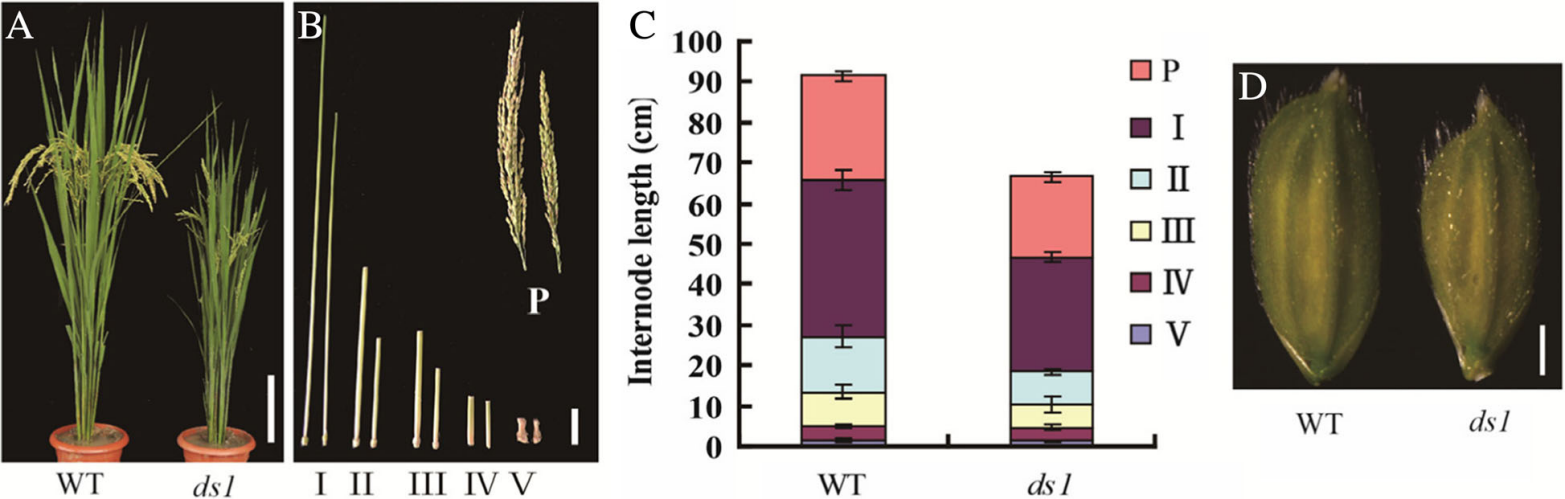
Phenotypic comparison of wild type and *ds1* mutant. **a** Gross morphology of the wild type and *ds1* plants. Scale bar, 10 cm. **b** Comparison of internode and panicle lengths between WT and *ds1*. Scale bar, 2 cm. **c** Statistical data for internode lengths described in (**b**). Data are presented as means ± SD. **d** Young spikelet hulls of WT (left) and *ds1* (right) plants. Scale bar, 1 mm

To identify genetic factors regulating rice architecture, we screened our Nanjing 35 radiation mutagenesis population by isolating dwarf mutants. A mutant was identified with dwarfism and decreased leaf angles, *ds1* (Fig. 1a-d).

**Fig. 2:**
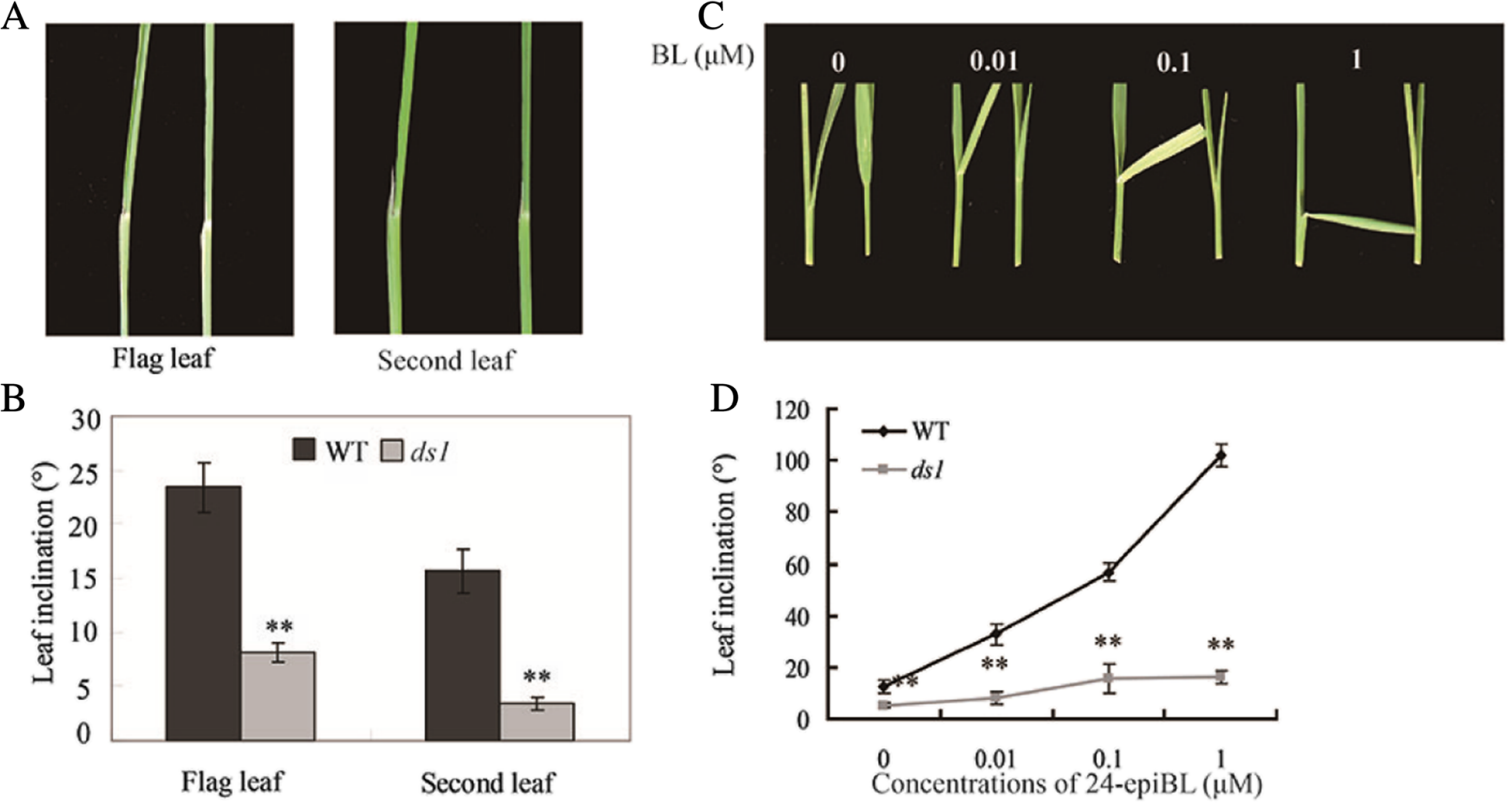
*DS1* is a positive regulator of BR signaling in rice. **a** Flag and second leaf angles of WT (left) and *ds1* (right) at maturity. **b** Statistical data for flag and second leaf angles shown in (**a**). Data are presented as means ± SD. **c** Lamina joint bending response to various amounts of 24-epiBL in WT (left) and *ds1* mutant (right) seedlings. **d** Statistical data for lamina joint bending angle assay described in (**c**). Data are means ± SD.**, significant difference between WT and *ds1* at *P* = 0.01

Differences between wild type and *ds1* mutant plants became apparent only after internode elongation when reduced height and darker leaf colour of the mutant were expressed. There was no difference between *ds1* and WT in young seedlings. Leaves of the mutant remained erect until maturity (Fig. 1a). Spikelet number per panicle, numbers of primary and secondary panicle branches of *ds1* were less than the WT (Additional file 1: Table S1). The panicles of *ds1* at maturity were almost erect whereas the panicles of WT clearly bend down. All internodes of *ds1* were shorter than those of WT (Fig. 1b-c). The dwarfism of *ds1* belonged to the dn type based on the classification of Yamamuro et al. (2000). Flag and second leaf angles of *ds1* were smaller than WT (Fig. 2a-b). These altered morphologies of *ds1* suggested that *DS1* participated in rice growth and development.

### The *ds1* mutant is less sensitive to BRs

As *ds1* had dwarfism, erect and dark green leaves, characteristic phenotypes of BR-related mutants, we hypothesized that *DS1* was involved in the BR pathway. Leaf angle in rice was known to be sensitive to the concentration of BL or related compounds (Wada et al. 1981). We tested the sensitivity of *ds1* to 24-epiBL in a lamina joint bending experiment. BL treatment caused a dose-dependent lamina joint inclination in WT, whereas *ds1* plants were insensitive. Treatment with BL showed an increased level leaf angle in WT plants, but little change in *ds1* plants (Fig. 2c-d).

### *DS1* regulates plant height and leaf angle by promoting cell elongation

**Fig. 3:**
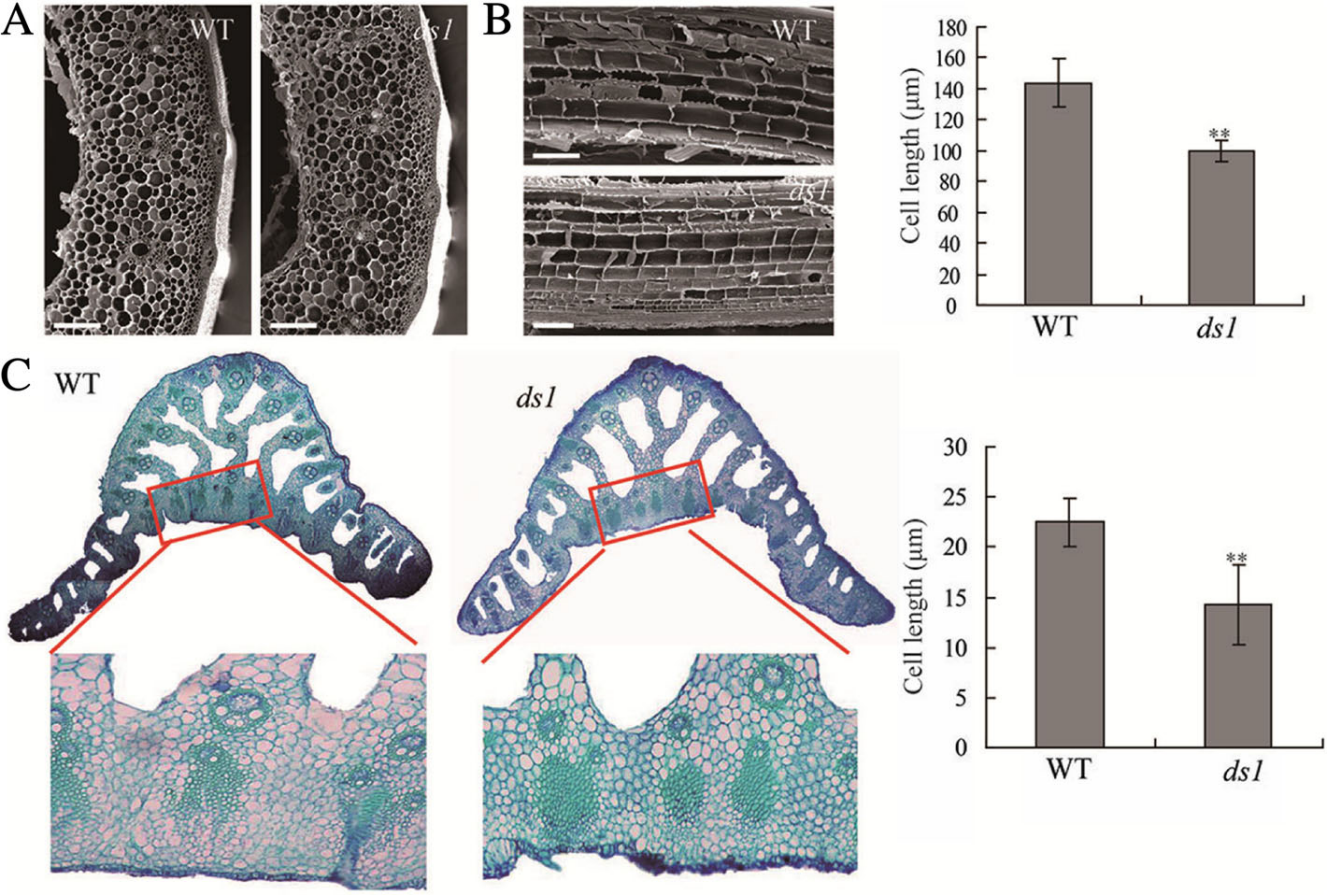
Histological analysis of the 2nd internodes and lamina joints. **a** Transverse sections of WT and *ds1* mutant 2nd internodes. Bar, 100 μm. **b** Longitudinal sections of WT and *ds1* mutant 2nd internodes. Bar, 500 μm.** indicates significant difference at *P* < 0.01. **c** Cross section of lamina joints of flag leaves in WT (left) and *ds1* (right). ** indicates significant difference at *P* < 0.01

To investigate the mutant phenotype of *ds1* at a cellular level we performed scanning electron microscopy (SEM) on *ds1* plants along with the WT (Fig. 3a-c). Dwarfism of *ds1* plants was mainly due to reduced cell length as measured in 2nd internodes (Fig. 3a-b). Observations on adaxial cells, which influence leaf angle, showed that those cells were also shorted in the *ds1* mutant (Fig. 3c). These results indicated that reduced cell length caused the dwarf phenotype of *ds1*.

As the cell wall is the major factor restricting cell elongation, many genes involved in cell wall synthesis are reported to regulate cell elongation (Ning et al. 2011). We investigated the expression levels of cell wall synthesis-related genes in WT and the *ds1* mutant. Expression levels of *IRX10L*, *CESA6*, and *UGA4e* were significantly down-regulated in *ds1* compared to WT (Additional file 1: Figure S1). Consistent with the morphological observations, this indicated that the dwarfism in *ds1* was caused by reduced cell length. To eliminate the possibility of decreased cell size in *ds1*, we analysed the expression levels of nine cell expansion regulators in WT and the *ds1* mutant (Additional file 1: Figure S1). Seven genes were significantly down-regulated in *ds1*, indicating that cell expansion was reduced. These results further confirmed that reduced cell elongation was the cause of the phenotype of *ds1*.

### Map-based cloning and functional analysis of *DS1*

To determine the gene responsible for the *ds1* phenotype, we made a cross between *ds1* and the wild type. F_1_ plants showed the WT phenotype and the F_2_ segregation ratio was consistent with that expected a single locus (χ^2^_3:1_ = 0.98, *P* > 0.05). These results indicated that *ds1* phenotype was due to a single recessive gene.

**Fig. 4:**
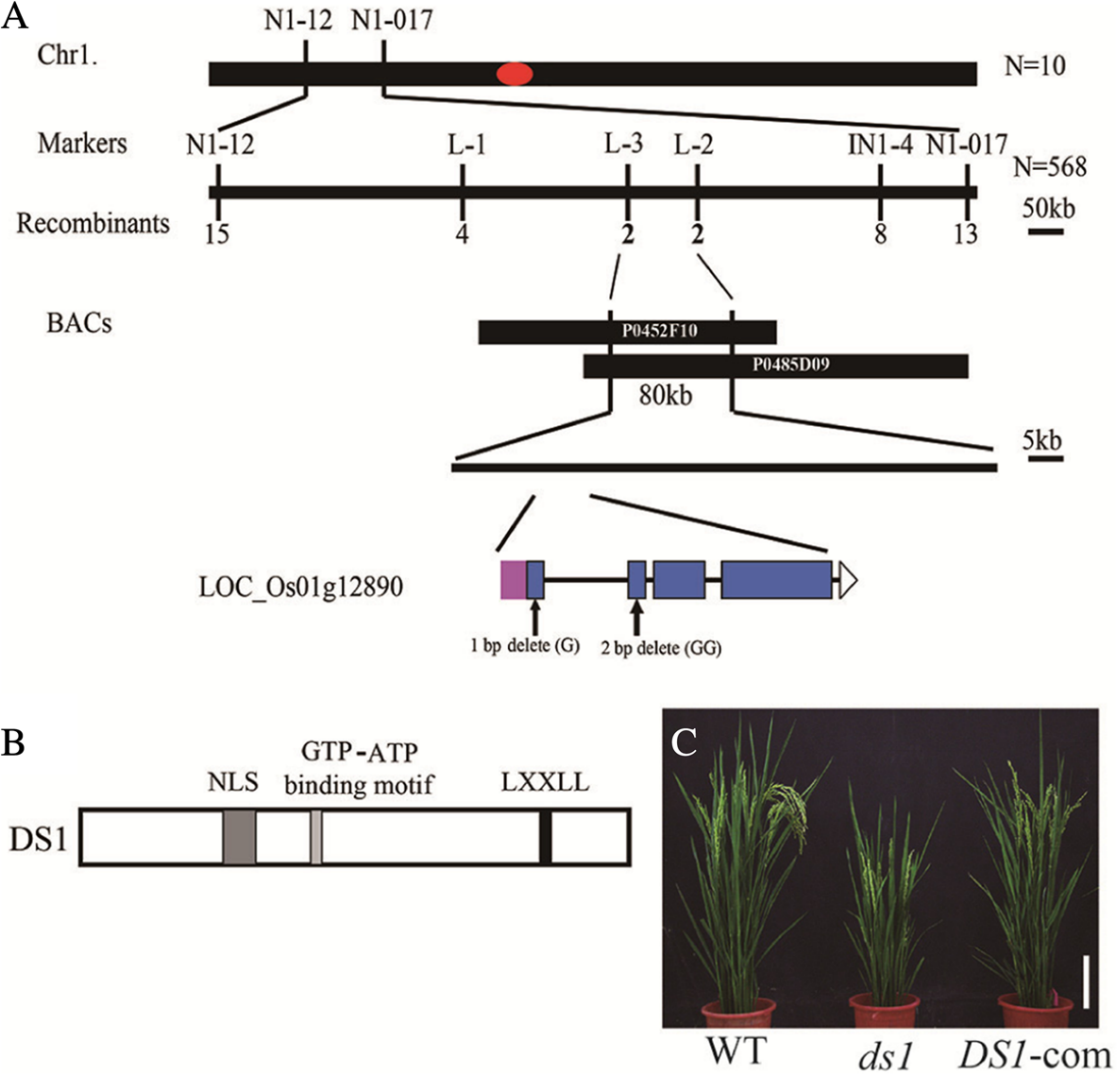
Map-based cloning of *DS1*. **a** Fine mapping of the *DS1* gene on chromosome 1. The *DS1* locus was mapped to an 80 kb region that contained 11 predicted open reading frames (ORFs). **b** DS1 protein structure. Gray box, NLS; light gray box, GTP-ATP binding motif; black box, LXXLL motif. **c** Characterization of T_1_ transgenic plants when *ds1* was transformed with the introduction of *DS1* fragment. Bar, 10 cm

To map the *DS1* locus, we next generated an F_2_ population from a cross between *ds1* and *indica* cultivar 93–11. *DS1* was roughly mapped on the short arm of chromosome 1 between SSR markers N1–12 and N1–017. We then used 568 recessive individuals from the same F_2_ population and narrowed down the *DS1* locus to a 80 kb region between markers L-3 and L-2 within BAC clones P0452F10 and P0485D09. A total of 11 putative genes were localized in the 80 kb region according to the genome annotation of Nipponbare (http://www.gramene.org/). On comparison of the mapped genomic region between WT and *ds1*, we detected a single nucleotide deletion at the first exon and two nucleotide deletions in the second exon of *LOC_Os01g12890* (Fig. 4a). We identified three domains in the DS1 polypeptide, including a nuclear localization signal (NLS), an ATP/GTP binding motif, and an LXXLL motif (Fig. 4b). The LXXLL motif had been demonstrated to mediate interactions that can activate or repress transcription (Heery et al. 1997; Torchia et al. 1997; Calonje et al. 2008).

The *OsEMF1* gene, annotated as the *LOC_Os01g12890* gene model, encodes an EMF1-like protein, involved in H3K27me3-mediated epigenetic silencing. In *Arabidopsis*, *EMBRYONIC FLOWER 1* (*EMF1*), a plant-specific protein, participated in H3K27me3-mediated silencing of target genes by acting downstream of *EMF2* and likely interacted with both PRC1 RING-finger proteins and PRC2 component *MULTICOPY SUPRESSOR OFIRA1* (*MSI1*) (Aubert et al. 2001; Calonje et al. 2008). In rice, *OsEMF1* plays an important role in palea development through maintaining H3K27me3-mediated epigenetic repression on *OsMADS58* (Zheng et al. 2015; Yan et al. 2015). To confirm whether mutation of *DS1* was responsible for the *ds1* phenotype, a CDS fragment of 3174 bp containing the entire *DS1* coding sequence and the *DS1* promoter were cloned into the pCUbi1390 binary vector, and the complementation construct was then transformed into the *ds1* mutant. All 8 independent *DS1*-complementation lines rescued normal leaf angle and plant height of *ds1* plants (Fig. 4c). These genetic evidence and transformation results confirmed that *DS1* was a newly identified *OsEMF1* allele, which influenced leaf angle and plant height. Homology analysis found that amino acid sequence of DS1 had 37% similarity and 20% homology to *Arabidopsis thaliana* EMF1 (AtEMF1) (Additional file 1: Figure S2).

### Expression pattern of *DS1*

**Fig. 5:**
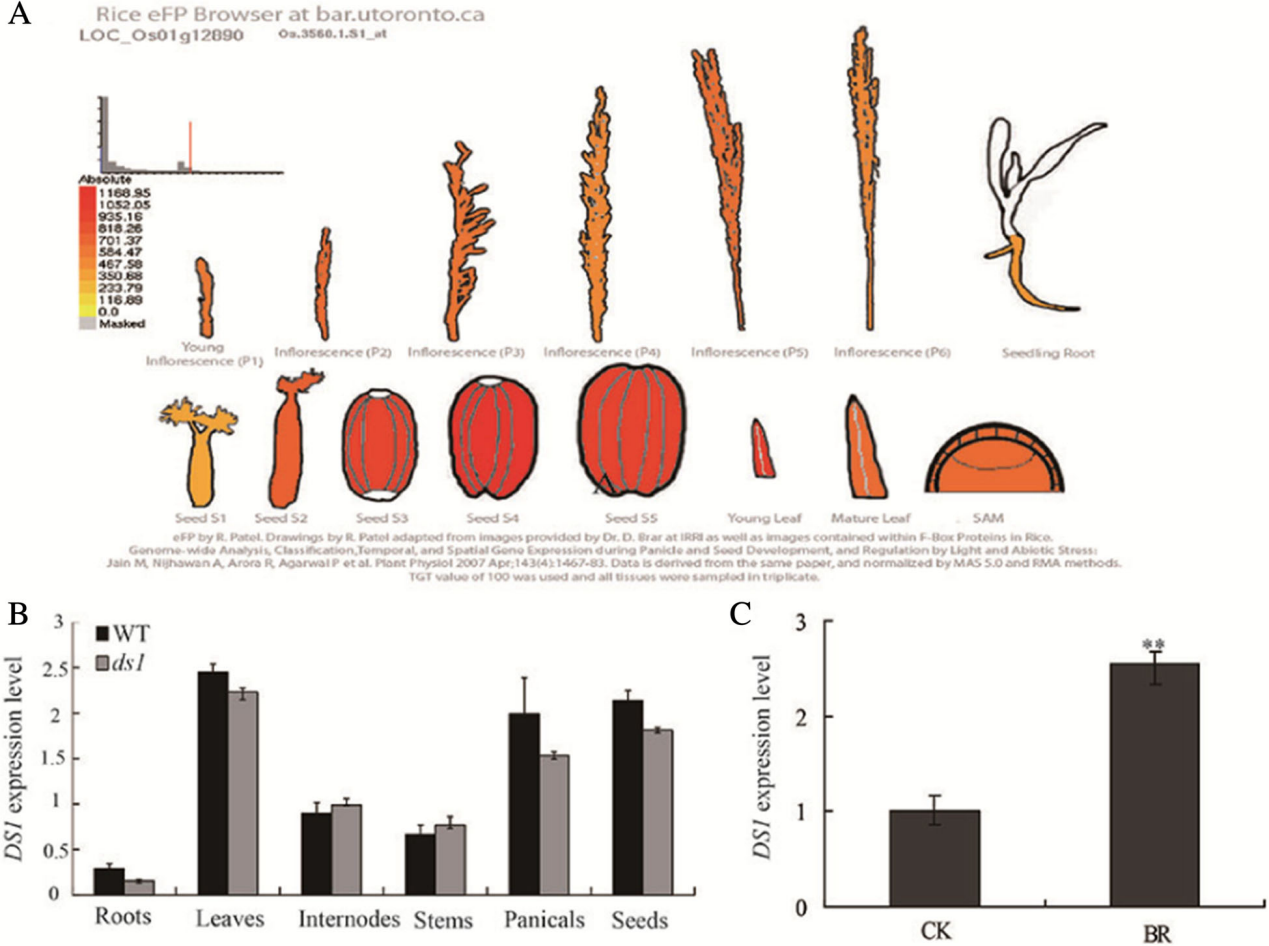
Spatiotemporal expression of *DS1.* **a** Expression pattern of *DS1* based on the Rice eFP Browser. **b** Quantitative RT-PCR analysis of *DS1* expression in various rice tissues. Roots and leaves were harvested from plants at the early booting stage. Internodes and stems were collected from 5-week-old seedlings. Panicles were collected when they had reached 2 cm length. Seeds were collected at grain filling. **c** Effect of 24-epiBL on *DS1* expression. 5-day-old seedlings of the wild type (WT) were harvested after treatment with 1 μM 24-epiBL. CK represents the mock-treated control

To further explore the biological functions of *DS1*, we found that *DS1* was highly expressed in the seeds,young leaves and panicles according to the Rice eFP Browser (http://bar.utoronto.ca/efprice/, Fig. 5a). Then we tested the *DS1* expression pattern in various organs from WT and *ds1* by quantitative real-time PCR. The results showed that *DS1* was highly expressed in young leaves, panicles and seeds, with the lowest expression in the roots. In comparison with the WT, the expression level was not significantly different in all tested organs of the *ds1* mutant (Fig. 5b). To determine whether the expression of *DS1* was affected by BR, seeds were cultured on half-strength medium containing 1 μM 24-epiBL and expression of *DS1* was determined by quantitative real-time PCR. As shown in Fig. 5c, the *DS1* expression was induced by BL treatment in WT.

### DS1 interacts with OsARF11

**Fig. 6:**
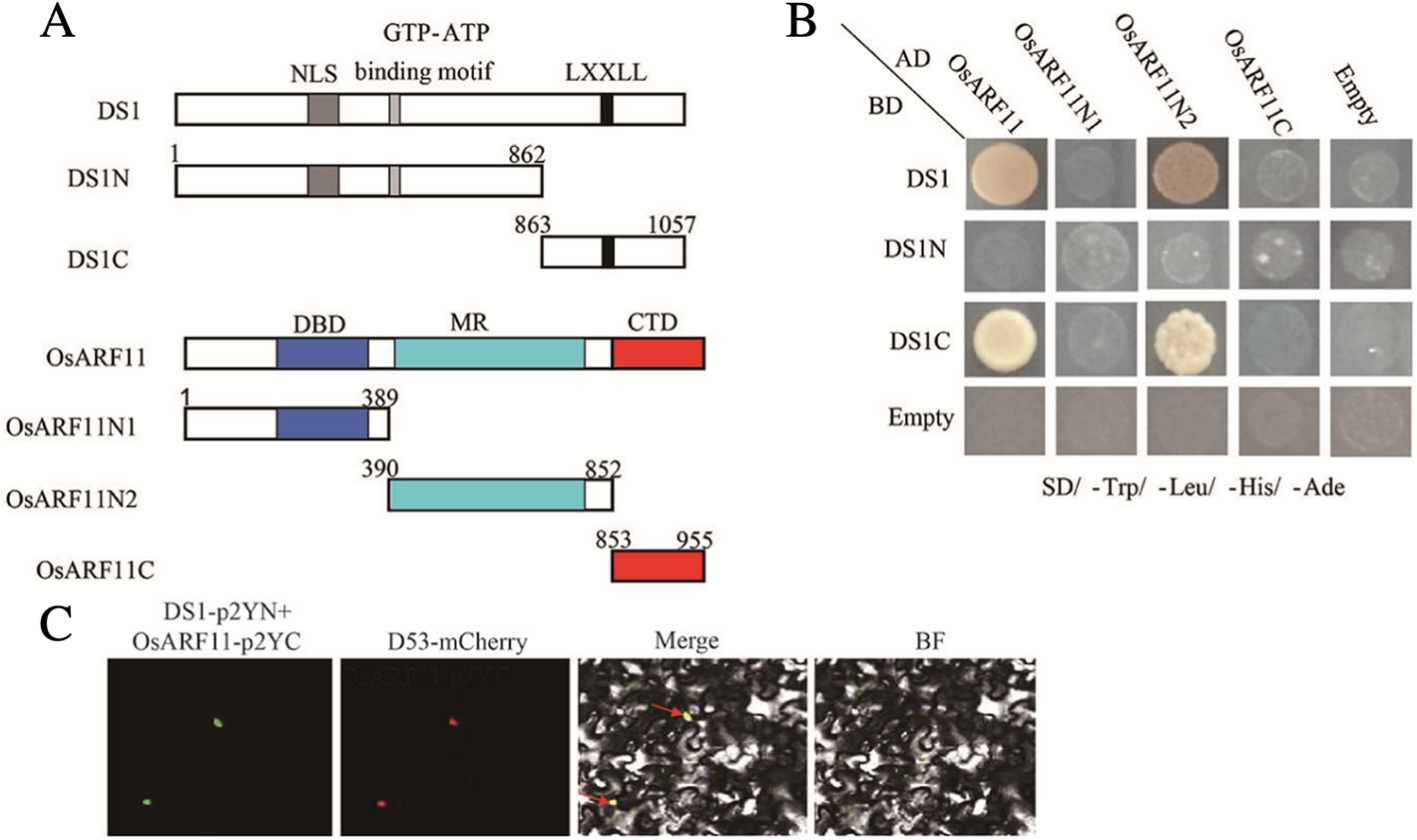
DS1 physically interacts with OsARF11. **a** Sketches showing the domain structures of DS1 and OsARF11 and various deletions. **b** Yeast two-hybrid assays showing the interactions between DS1, OsARF11, and their derivatives. Transformed yeast cells were grown on SD/−Trp/−Leu/-His/−Ade. **c** BiFC assay showing DS1-YFP^N^ and OsARF11-YFP^C^ interaction to form a functional YFP in the nucleus

To gain a deeper insight into the molecular function of DS1, we performed a yeast two-hybrid (Y2H) screening using DS1 as bait. We obtained many candidate interacting proteins, including auxin response factor 11 (OsARF11). The interaction between DS1 and OsARF11 was further confirmed using the full-length cDNA in yeast (Fig. 6a-b). Auxin response factors (ARFs), including *OsARF11* and *OsARF19*, were reported to regulating plant height and leaf angle. Loss-of-function *osarf* mutants showed decreased leaf angle and dwarfism, similar to the *ds1* mutant. Also, OsARFs directly regulate expression of *OsBRI1* by directly binding to the AuxRE element in the promoter of *OsBRI1* (Shen et al. 2010; Sakamoto et al. 2013; Zhang et al. 2015a).To validate this interaction, we performed a bimolecular fluorescence complementation (BiFC) study. The N-terminal half of YFP was fused to DS1, and the C-terminal half of YFP was fused to OsARF11. Both constructs were then transiently expressed in tobacco (*Nicotiana bethamiana*). YFP fluorescence was observed in the nucleus, confirming that DS1 interacted with OsARF11 in planta (Fig. 6c). Taken together, these results indicated that DS1 might regulate plant height and leaf angle through interaction with OsARF11.

### Expression pattern and subcellular localization of *OsARF11*

**Fig. 7:**
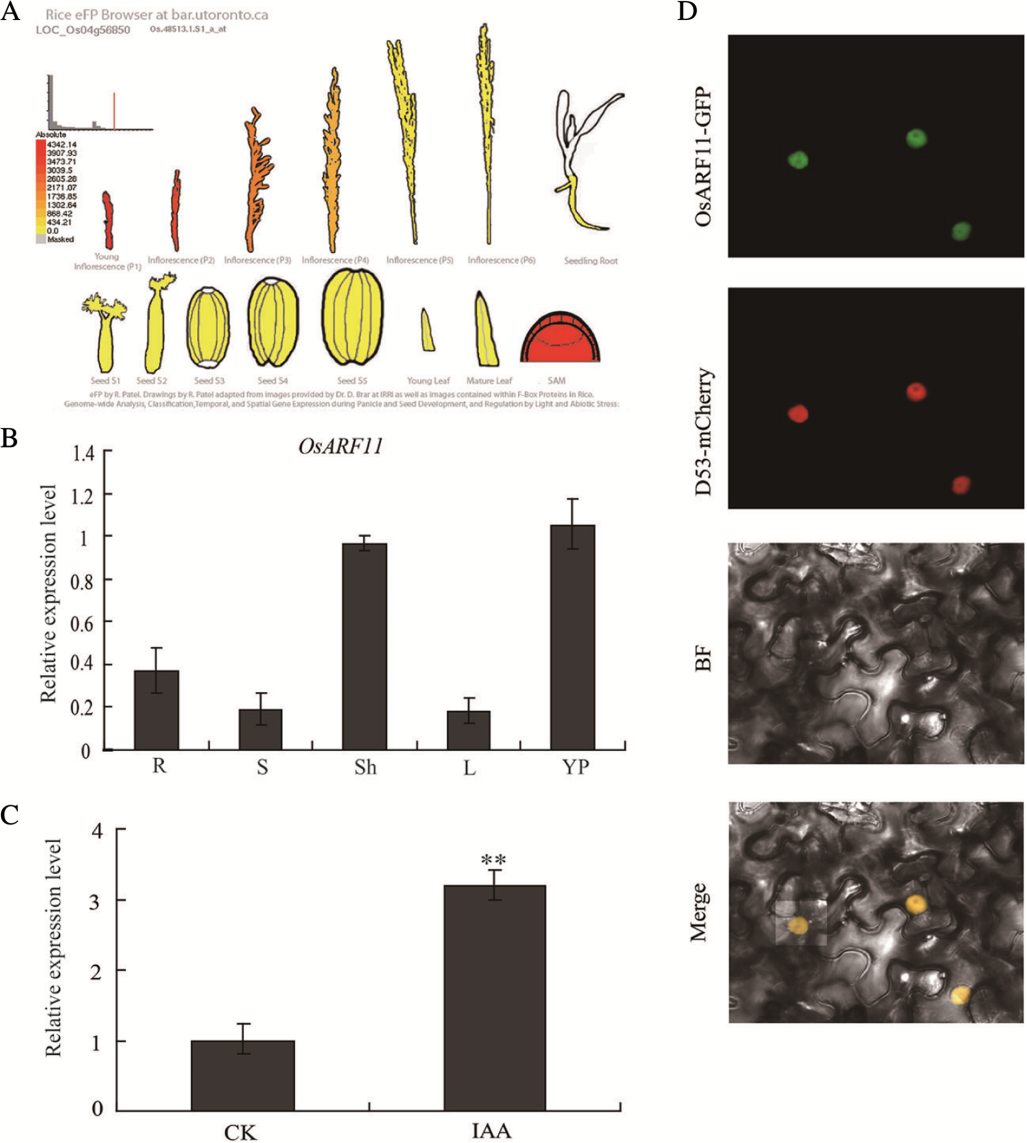
Expression pattern and subcellular localization of OsARF11. **a** Expression pattern of *OsARF11* based on the Rice eFP Browser. **b** Expression pattern of *OsARF11* detected by qRT-PCR. The experiment was repeated three biological times with similar results. Error bars represent standard deviations (SD) (*n* = 3). R, root; S, stem; L, leaf; YP, young panicles; Sh, Leaf sheath. **c** *OsARF11* expression under auxin treatments. One-week-old WT seedlings were grown in solution containing 10 μM IAA for 4 h. ** indicate significant difference at *P* < 0.01. Error bars represent the SD (n *=* 3). **d** Subcellular localization of the OsARF11-GFP fusion protein in tobacco epidermal cells

To verify whether the expression pattern of *OsARF11* is similar to that of *DS1*, we found that *OsARF11* was highly expressed in the shoot apical meristem and young panicles according to the Rice eFP Browser (http://bar.utoronto.ca/efprice/, Fig. 7a). We employed a quantitative RT-PCR (qRT-PCR) assay to examine OsARF11 expression in wild-type plants. Real time qPCR analysis showed that OsARF11 transcripts accumulated in all examined tissues, including sheaths, young panicles, roots, stems and leaves (Fig. 7b). These results indicated that *OsARF11* showed a very similar expression pattern to *DS1*. We further, found that *OsARF11* expression was induced by auxin treatment (Fig. 7c), suggesting that its function might be regulated by auxin. We next examined the subcellular localization of OsARF11 protein. A construct harboring a OsARF11-GFP fusion was introduced into tobacco epidermal cells by Agrobacterium. Fluorescent signals of OsARF11-GFP fusion protein were observed exclusively in the nucleus of tobacco cells (Fig. 7d), indicating that OsARF11 is a nuclear protein, consistent with the function of OsARF11.

### DS1 and OsARF11 co-regulate *D61*/*OsBRI1* expression

**Fig. 8:**
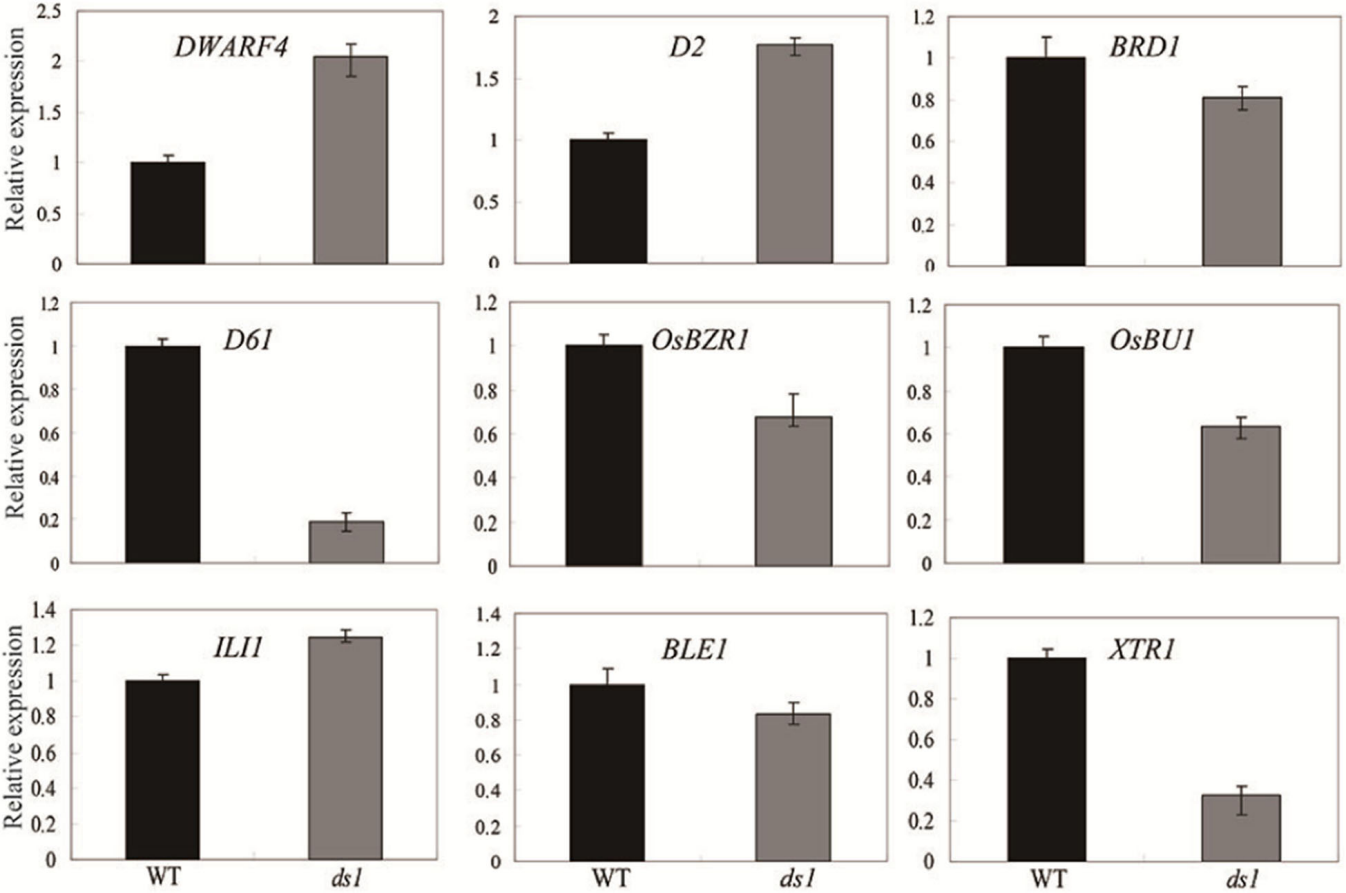
Expression of BR biosynthesis and signal transduction genes. Real-time PCR analysis of BR biosynthesis and signal transduction genes in the wild type and *ds1* seedling at 15 days after germination. Data are presented as means ± SD (n = 3)

As shown in Fig. 2c, the *ds1* mutant was insensitive to BL treatment. We investigated the effect of *DS*1 on the expression of BR-related genes and the results showed that disruption of *DS1* led to significantly increased expression of *D2* and *DWARF4*, two important brassinosteroid biosynthesis rate-limiting enzymes (Hong et al. 2003; Sakamoto et al. 2006). By contrast, *DS1* had large effects on expression of BR signal genes, including *D61*/*OsBRI1*, *OsBZR1*, *OsBU1* and *XTR1* (Fig. 8). This indicated that *DS1* could be involved in BR signaling.

**Fig. 9:**
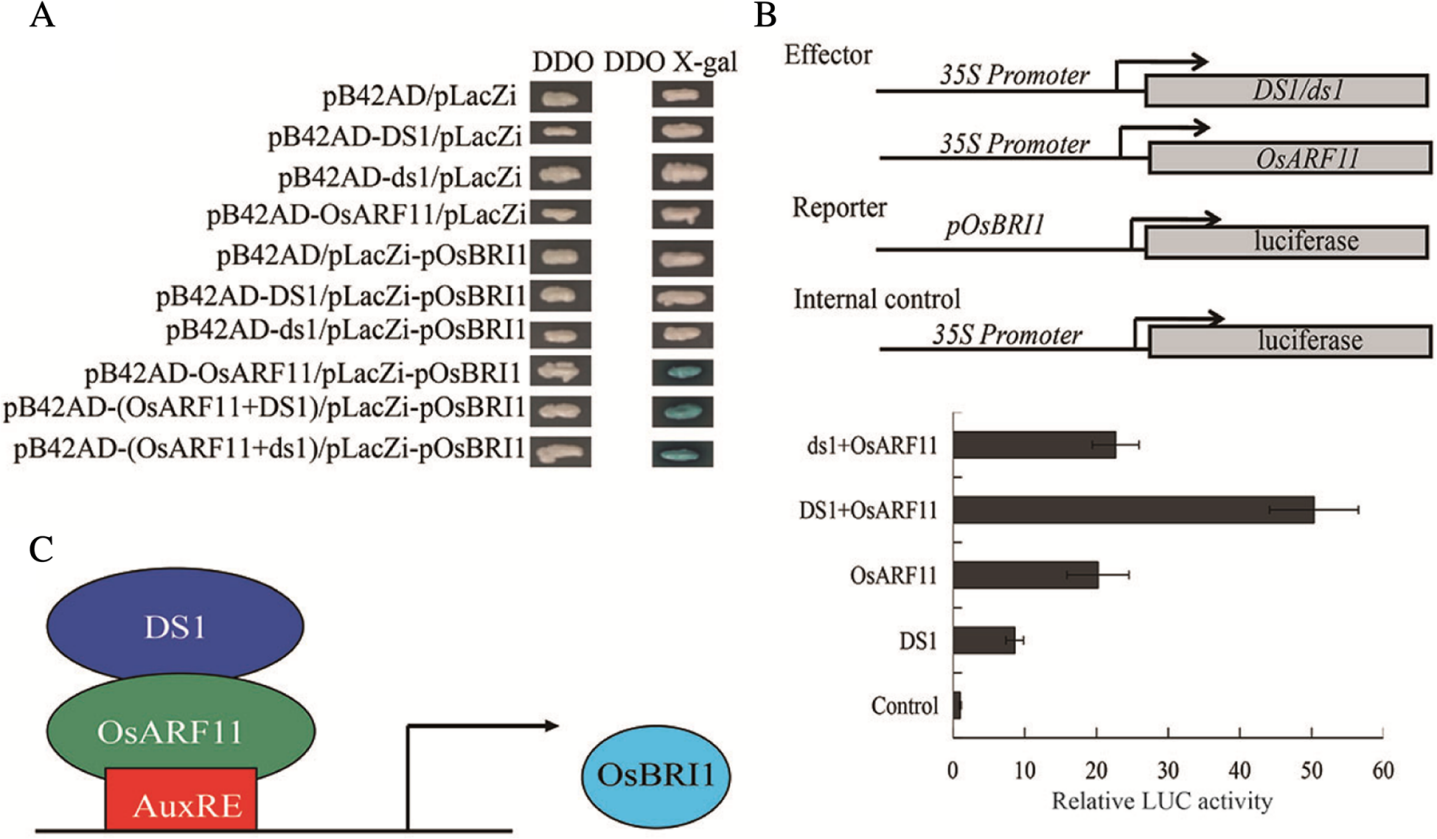
DS1 interacts with OsARF11 to promote the expression of *OsBRI1*. **a** Yeast one-hybrid (Y1H) analysis of DS1, OsARF11 and *OsBRI1* promoter. The bait vector containing the *OsBRI1* promoter fragment-fused lacZ reporter gene, and the prey vector containing DS1/OsARF11-fused GAL1 activation domain were co-transformed into yeast cells (EGY48). **b** Transient expression assays of *OsBRI1* transcriptional activity modulated by DS1/ds1 and OsARF11 in rice protoplasts. Various constructs used in transient expression assays are shown in the upper panel. p*OsBRI1*:LUC was cotransformed with either the effector or empty vector (control) into rice protoplasts. Relative LUC activity indicating the level of *OsBRI1* expression activated by the effector is shown in the lower panel. Values are means ± SD of three biological replicates. **c** Model representing mechanism of action of DS1 in regulating *OsBRI1*

Previous studies showed that *OsARF11* promotes plant growth and leaf angle by specific binding to an auxin response element (AuxRE) in the promoter of *OsBRI1* (Sakamoto et al. 2013), and that OsARF19 binds to an AuxRE element in the promoters of *OsBRI1* and *OsGH3–5* (Zhang et al. 2015a). We analyzed the promoters of four BR signal genes (*OsBRI1*, *OsBZR1*, *OsBU1* and *XTR1*), and found that only the promoter of *OsBRI1* contained the auxin response element. To confirm the binding of DS1 or OsARF11 to the *OsBRI1* promoter, we performed yeast one hybrid assays using in vitro-expressed DS1 and OsARF11. As shown in Fig. 9a, OsARF11 bound to the *OsBRI1* promoter but DS1 failed to bind the promoter of *OsBRI1*.

To examine the regulation of DS1 and OsARF11 on the expression of its target gene *OsBRI1*, we performed transient expression assays using ~ 2 kb of the *OsBRI1* promoter fused to the LUC gene as a reporter. Effector constructs for OsARF11 were expressed under control of the 35S promoter and transfected together with the reporter construct into rice protoplasts. Higher LUC activity was detected when OsARF11 protein was transfected with the reporter construct compared with the internal control (Fig. 9b). These findings strongly support the hypothesis that DS1 and OsARF11 coregulate *OsBRI1* in controlling rice plant architecture.

## Discussion

Plant architecture is a major factor underlying grain yield and determination of planting density and photosynthesis efficiency in rice. The molecular mechanisms of plant architecture have been extensively studied and many critical genes have been identified. Most of these genes are involved in the Auxin and BR pathways. Auxin and BR signaling share many transcriptional target genes in regulating rice plant architecture (Yin et al. 2002; Nemhauser et al. 2004), suggesting that interactions between TFs involved in auxin and BR signaling might act as the points of auxin–BR crosstalk, but only a few examples have been reported. One is the response of *SAUR15* to auxin and BR. *SAUR15* expression depends on the simultaneous interaction of BES1 and ARF5 within the promoter of the S*AUR15*, gene that produces a hormone with an Up at Dawn (HUD)-type E-box and AuxRE cis elements (Walcher and Nemhauser 2012). Another example is that BZR1 and ARF6 were shown to interact and regulate a large number of common target genes (Oh et al. 2014).

In this study, we characterized mutant *ds1*, which showed dwarfing and a reduced leaf angle compared to the WT. Through map-based cloning and genetic transformation, *DS1* was identified as a new allele of *OsEMF1*. In rice, *OsEMF1* functions in influencing the level of the H3K27me3 at the *OsMADS58* locus, and repressing the expression of *OsMADS58* to control palea development (Zheng et al. 2015; Yan et al. 2015). *ds1* became less sensitive to BL treatment relative to the wild type in a lamina joint inclination test. We found that the expression level of *OsBRI1* was down-regulated 5 times in the *ds1* mutant. *OsEMF1* inhibits the expression of target genes by mediating H3K27me3, which is not consistent the down-regulation of *OsBRI1* expression in the *ds1* mutant. *DS1* may influence the expression of *OsBRI1* through histone methylation or other unknown mechanisms. We demonstrated that DS1 physically interacted with OsARF11 to positively co-regulate expression of *OsBRI1*. Indeed, *DS1* and *OsARF11* showed similar expression profiles.

Following the initial discovery of the effect of auxin-BR crosstalk on lamina joint bending, several genes have now been reported to be involved (Luo et al. 2016). For instance, *LPA1* negatively regulates lamina joint bending by inhibiting BR signaling and increasing the expression of auxin transporters (Liu et al. 2016). In a recent study, the SMOS1-SMOS2/DLT complex positively regulated expression of *OsPHI-1*, and treatment of *smos* mutants with auxin and BR still showed a synergistic effect on lamina joint bending (Hirano et al. 2017). In the current study, we showed that DS1-OsARF11 is invoved in auxin-BR crosstalk in rice. It is plausible that lamina joint bending is determined by a transcriptional network consisting of DS1-OsARF11, SMOS1-SMOS2/DLT, LPA1, and other factors regulated by auxin and BR signaling.

In high-density plantings, characteristic semi-dwarf phenotypes with erect leaves are ideal phenotypes for improving grain yield (Lu et al. 2013; Wu et al. 2008). Further, identification of other downstream genes of *DS1/OsEMF1* will be needed for fully understanding the network regulating plant growth and development. In addition, the BR signaling pathway is necessary for auxin function in controlling leaf angle, indicating that the roles of auxin and BR are interdependent. Our study uncovers a previously unknown regulatory factor in plant architecture and the coordinating network of auxin and BR signaling. These findings might be useful for manipulating optimal rice architectures.

## Conclusions

In this study, we found that DS1/OsEMF1 functions in BR signaling by interacting with OsARF11. The underlying mechanisms might be useful for engineering rice architecture to increase rice yield.

## Methods

### Plant material and growth conditions

The *ds1* mutant obtained from an irradiated population of *japonica* variety Nanjing 35 was stably inherited. The mutant was self-pollinated for several generations until shown to be heritable. Crosses were made between the *ds1* mutant and variety Nanjing 35 for genetic analysis. 93–11 (*indica*) was employed as a control in mapping of *DS1*. Pre-germinated seeds were sown in nursery beds in the field and the 1-month-old seedlings were transplanted into a paddy field in Nanjing.

### Trait measurement

The grain length, width, and thickness were measured by an electronic digital display vernier caliper and fully filled grains were used for measuring the 1000-grain weight. The plant height, primary and secondary branch number, panicle length, and grain number per panicle were obtained from the measurement of the main stem. Numbers of tillers per plant were recorded at the time of harvest. Three biological repeats were measured for each sample.

### Histological analysis

The second internodes and lamina joints of the flag leaves were harvested at heading date, respectively. All samples fixed in glutaric dialdehyde for 48 h. The fixed internode samples were soaked in 2% (*w*/*v*) OsO_4_ for 2 h, dehydrated in a graded ethanol series (70, 80, 90, 100, and 100%), cleared in a xylene series, and then sputter coated with platinum. Second internode sections were observed by SEM (HITACHI, S-3000 N). Fixed lamina joints were embedded in paraplast. After sectioning, 10 μm thick sections were dewaxed with xylene, rehydrated, stained with 1% toluidine blue, and observed with a Leica DM5000B microscope. Cell lengths of each organ were measured with IMAGEJ software.

### Lamina joint bending assay

Brassinolide (BL) was dissolved in ethanol. For BL treatment, sterile water was added to reach final concentrations of 0.01, 0.1, and 1 μM. Germinated seedlings of WT and *ds1* were grown in the light for 10 d at 30 °C. Lamina joint segments were floated on distilled water for 72 h containing various concentrations of brassinolide in the light. The lamina joint bending angles were then photographed and measured. Twenty plants were used for each treatment.

### Genetic analysis and map-based cloning of *DS1*

Populations for genetic analysis and mapping were from crosses between *ds1* and *japonica* cv. Nanjing 35 and *indica* cv. 93–11. For mapping of *DS1*, 7680 F_2_ plants were generated from a cross between *ds1* mutant and *indica* cv. 93–11, and 568 individuals with the clear *ds1* phenotypes were selected for mapping. Additional molecular markers were designed from comparative sequences of Nipponbare and 93–11. Molecular markers chosen for mapping are listed in Additional file 1: Table S2. The PCR procedure was: 95 °C for 5 min, followed by 33 cycles of 95 °C for 30 s, annealing for 30 s, 72 °C for 40 s and a final elongation step at 72 °C for 5 min. All the populations were grown in the field at Nanjing in Jiangsu province, and Lingshui County in Hainan province.

### Complementation of the *ds1* mutant

To test whether the mutation in *LOC_Os01g12890* was responsible for *ds1*, an ~ 2 kb promoter and full length cDNA of *DS1* were cloned into the pCUbi1390 binary vector (primer sequences are listed in Additional file 1: Table S3). The resulting binary plasmid was then introduced into *Agrobacterium tumefaciens* strain EHA105. Calli induced from homogeneous *ds1* seeds were used for transformation. The transgenic plants were grown in a glasshouse to observe the phenotypes.

### RNA extraction and qRT-PCR analysis

Total RNAs were isolated from various plant organs using Trizol reagent (Invitrogen) according to the manufacturer’s instructions. First strand cDNAs were synthesized from 2 μg total RNA using a PrimeScript 1st Strand cDNA Synthesis Kit (Takara, Dalian).

For qRT-PCR, SYBR Premix *Ex* TaqTM kit (Takara) was added to the reaction system and run on an ABI Prism 7500 real-time PCR system and the *OsActin* gene was used as an internal control. The relative expression level was calculated by 2^-ΔΔCt^. The primers used for BR-related genes and cell wall synthesis-related genes are listed in Additional file 1: Table S3. The PCR procedure was: 95 °C for 30 s, followed by 40 cycles of 95 °C for 5 s, 60 °C for 34 s.

### Yeast two-hybrid assay

For yeast two-hybrid analysis, various fragments were cloned into pGBKT7 and pGADT7 to construct BD-DS1/N/C/ and AD-OsARF11/N1/N2/C/, respectively. The reported gene assay was performed following the manufacturer’s instructions (Clontech). Yeast transformation and screening procedures were performed according to the manufacturer’s instructions (Clontech). The yeast cells were transformed with the rice cDNA library constructed from young panicles of the japonica cultivar “Nipponbare”. Positive transformants were screened for growth on SD/−Trp/−Leu/-His/−Ade. The yeast clones were cultured and sequenced. The primers used for yeast two-hybrid analysis were listed in Additional file 1: Table S3.

### BiFC assay

The full-length cDNA of DS1 was cloned into the p2YN vector to construct the DS1-YFP^N^ fusion protein. OsARF11 was cloned into the p2YC vector to produce an OsARF11-YFP^C^ fusion protein, (primer sequences are listed in Additional file 1: Table S3). BiFC analyses were performed in tobacco, as described previously (Waadt and Kudla 2008). The primers used for BiFC assay are listed in Additional file 1: Table S3.

### Transient expression and yeast one-hybrid assays

To generate the p*OsBRI1*:LUC reporter construct, ~ 2 kb of the *OsBRI1* promoter was cloned into pGreenII-0800-LUC. *OsARF11* and *DS1* full length cDNA were cloned into the modified pAN580 binary vector, respectively. The construct vectors were co-transformed into rice protoplasts. Rice protoplasts were prepared, transfected, and cultured as previously described (Wang et al. 2016). The luciferase activity assay was calculated following the manufacturer’s instructions (Promega) and the data presented are the averages of three biological replicates.

For yeast one-hybrid (Y1H) assays, the full length cDNA of *DS1*, *OsARF11* and promoter of *OsBRI1* were amplified using gene-specifc primers (Additional file 1: Table S3), and the amplifed products were cloned into the pB42AD vector and reporter plasmid pLacZi, respectively. Plasmids were co-transformed into yeast strain EGY48 according to the manufacturer’s manual (Clontech).

## Additional file

Additional file 1: **Table S1** Comparison of agronomic traits between WT and *ds1.* **Table S2** Primers used in mapping. **Table S3** Primers used in real-time PCR and vector construction. **Figure S1** Relative expression levels of cell wall synthesis-related genes (*CESA6*, *IRX10L*, *GT8*, *UGA4e*, and *CSLF6)* and cell expansion-related genes, *Exp1*, *ExpA8*, *ExpA10*, *ExpA17*, *ExpA30*, *ExpB2*, *ExpB3*, *ExpB5*, and *ExpB12*. **Figure S2** Alignment amino acid sequences of DS1 and AtEMF1. (DOC 1088 kb)

## Abbreviations

BiFC: Bimolecular fluorescence complementation
GFP: Green fluorescent protein
OsARF11: Auxin response factor 11
qRT-PCR: Quantitative real-time PCR
SEM: Scanning Electron Microscope
WT: Wild type
Y1H: Yeast one-hybrid
