# Supplementary material for: DS1/OsEMF1 interacts with OsARF11 to control rice architecture by regulation of brassinosteroid signaling
Source: Rice (N Y). 2018 Aug 6;11:46. doi: 10.1186/s12284-018-0239-9 (PMC6082143; doi:10.1186/s12284-018-0239-9)
Supplement: Supplementary file 1 — Table S1 Comparison of agronomic traits between WT and ds1. Table S2 Primers used in mapping. Table S3 Primers used in real-time PCR and vector construction. Figure S1 Relative expression levels of cell wall synthesis-related genes (CESA6, IRX10L, GT8, UGA4e, and CSLF6) and cell expansion-related genes, Exp1, ExpA8, ExpA10, ExpA17, ExpA30, ExpB2, ExpB3, ExpB5, and ExpB12. Figure S2 Alignment amino acid sequences of DS1 and AtEMF1. (DOC 1088 kb) [file 12284_2018_239_MOESM1_ESM.doc]

###### Additional files

###### Additional files Tables S3 and two figures

**TITLE:** DS1/OsEMF1 interacts with OsARF11 to control rice architecture by regulation of brassinosteroid signaling

**X. Liu^1*^• C.Y. Yang^1*^ • R. Miao^1^ • C.L. Zhou^1^ • P.H. Cao^1^ • J. Lan^1^ •X.J. Zhu^1^ • C.L. Mou^1^ • Y.S. Huang^1^ • S.J. Liu^1^ • Y.L. Tian^1^ • T.L. Nguyen^1^ • L.Jiang^1^**
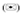
**• J.M. Wan^1,2^**
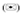


**^1^***State Key Laboratory for Crop Genetics and Germplasm Enhancement, Jiangsu Plant Gene Engineering Research Center, Nanjing Agricultural University, Nanjing 210095, China.*

**^2^** *National Key Facility for Crop Gene Resources and Genetic Improvement, Institute of Crop Science, Chinese Academy of Agricultural Sciences, Beijing 100081, China*

- Corresponding authors:

Ling Jiang

Telephone: +86-25-84399061

Fax: +86-25-84399061

E-mail: [jiangling@njau.edu.cn](mailto:jiangling@njau.edu.cn)

Jianmin Wan

Telephone: +86-25-84396516

Fax: +86-25-84396516

E-mail: wanjm@njau.edu.cn

**Table S1 Comparison of agronomic traits between WT and *ds1***

| Trait | WT | *ds1* | *P* value |
| --- | --- | --- | --- |
| Plant height (cm) | 91.35± 4.28 | 63.62± 6.11** | 2.18E-9 |
| Panicle length (cm) | 27.62 ± 1.66 | 21.16±0.23** | 1.32E-3 |
| Spikelet fertility (%) | 85.58±1.05 | 61.44±0.87** | 3.38E-4 |
| 1000- grain weight (g) | 25.31±0.25 | 17.99±0.57** | 1.43E-3 |
| Grain number per main panicle | 274.41±15.31 | 176.45±10.35** | 9.17E-6 |
| Primary branch number | 15.5± 3.71 | 11.5± 3.87** | 4.13E-4 |
| Grain length (mm) | 10.05±0.46 | 8.63±0.49** | 3.57E-8 |
| Grain width (mm) | 4.66±0.16 | 4.02±0.29** | 1.70E-4 |
| Grain thickness (mm) | 2.04±0.07 | 1.99±0.08 | 0.09 |
| Number of tillers | 6.9±1.35 | 6.4±1.25 | 0.22 |
| Secondary branch number | 63.5±5.53 | 42.8±4.78** | 9.45E-9 |

Data are averages of 20 samples ± SD, ** indicates a significant difference between WT and *ds1* at *p* = 0.01.

**Table S2 Primers used in mapping**

| Marker | Forward primer sequence (５′ →３′ ) | Revers primer sequence （５′ →３′ ） |
| --- | --- | --- |
| N1-12 | AAATGGGCTTCCTCCTCTTC | CAGCCTTGATCGGAAGTAGC |
| N1-017 | TGCCTACTCAGCAACTAACAC | ACTTTGCAGTTTGCACATC |
| IN1-4 | CAGCATACAGTACGCATCATCA | GCTCGTATCTCGATGAGTCCA |
| L-1 | CTGCCACCCAAACCAAATC | CAGCAATGAGGCGGACGA |
| L-2 | TCGGTGCTTGATGATGTTGAGGAA | TGGAGGAGTCGCCCACGAGTAG |
| L-3 | CAGCAAGCGGCACAATAC | TTGGCAATGCTAAGAAGA |
| DS1-CDS | ATGGAGATTGTTGCAGTAGA | TCAGCGGTAATCCATATACT |
| DS1-g | CAAATCCCTTCTTACCCTAT | CGTTTCAACCTTAACACCGA |

**Table S3 Primers used in real-time PCR and vector construction**

| Marker | Forward primer sequence (５′ →３′ ) | Revers primer sequence （５′ →３′ ） |
| --- | --- | --- |
| DS1 | TCTCTATCTCGGATTTTCCATGACC | AACCATCACTTGTGCCATTCTGCTT |
| OsActin | TCAGATGCCCAGTGACAGGA | TTGGTGATCTCGGCAACAGA |
| DS1 | TTGCTCGTGGTGGAACTGGT | GCCATTCTGCTTTGCGGGAA |
| DS1OE | TTACTTCTGCACTAGGTACCATGGAGATTGTTGCAGTAGA | GAATTCCCGGGGATCCTCAGCGGTAATCCATATACT |
| BD-DS1 | CATGGAGGCCGAATTCATGGAGATTGTTGCAGTAGA | GCAGGTCGACGGATCCTCAGCGGTAATCCATATACT |
| BD-DS1-N | TCCCCCGGGGATGGAGATTGTTGC | ACGCGTCGACTGGATCCATCATTC |
| BD-DS1-C | CGGGATCCGTTCAACATTAGCAAG | ACGCGTCGACTCAGCGGTAATCCA |
| OsARF11AD | GGAGGCCAGTGAATTCATGATGAAGCAGGCGCAGCA | CGAGCTCGATGGATCCTTACGCAGTATTCCAATACC |
| OsARF11AD-N1 | GGAGGCCAGTGAATTCATGATGAAGCAGGCGCAGCA | CGAGCTCGATGGATCCGTTAGCACGCCTGATTCCTA |
| OsARF11AD-N2 | GGAGGCCAGTGAATTCTGCTAACCGACAGCCAACTAA | CGAGCTCGATGGATCCGAATGTCCTCATCCTCTTTA |
| OsARF11AD-C | GGAGGCCAGTGAATTCGGACATTCACCAAGGTATAT | CGAGCTCGATGGATCCTTACGCAGTATTCCAATACC |
| p2YN-DS1 | AACGATAGTTAATTAAATGGAGATTGTTGCAGTAGA | ACCTCCTCCACTAGTTCAGCGGTAATCCATATACT |
| p2YC-OsARF11 | CATTTACGAACGATAGTTAATTAAATGATGAAGCAGGCGCAGCA | CACTGCCACCTCCTCCACTAGTTTACGCAGTATTCCAATACC |
| pB42AD-DS1 | TGCCTCTCCCGAATTCATGGAGATTGTTGCAGTAGA | CGAGTCGGCCGAATTCTCAGCGGTAATCCATATACT |
| pB42AD-OsARF11 | TGCCTCTCCCGAATTCATGATGAAGCAGGCGCAGCA | CGAGTCGGCCGAATTCTCATTCGAATTGTTCATATG |
| pLacZi-OsBRI1pro | ATCTGTCGACCTCGAGCGGCTGTCACCGTTACTCCT | GAGCACATGCCTCGAGCTTGTCTTGGTTTGCCCATT |
| DS1--PAN580-VP16 | CGGAGCTAGCTCTAGAATGGAGATTGTTGCAGTAGA | TTCTTCTTAGGCTGCAGGGATCCGCGGTAATCCATATACT |
| OsARF11--PAN580-VP16 | CGGAGCTAGCTCTAGAATGATGAAGCAGGCGCAGCA | TTCTTCTTAGGCTGCAGGGATTACGCAGTATTCCAATACC |
| OsBRI1-LUC | CCCAAGCTTGTACCCCTACTCCGTGATGCTCGAGGACGTGAA | AACTGCAGTATATATATAGTCCTCTCCACAAGTAAATGGCATCCGAAT |
| OsBRI1q | GCAAGGGTATCTGATTTCGGT | CAAGAGTGGACACGCTAAGGT |
| OsBZR1q | AGATGGTTCCTTTCGTGGAC | AGAATGAAATCGCCCAAATC |
| OsBU1q | CATCTCCAAGCTCCAGTCCCT | GCTCTTGATGTAGCTGCACGTCT |
| D2q | CCTTTTGGTGGTGGGCAGAG | TGGGGAAGTTGACGATGTGGT |
| OsDWARF4q | AGTCGCGTGCTGCCATTCTCGGAGTAATAG | AGCAAGCTCAGCAAGAGGTCCAGGATTTGC |
| ILI1q | GATCTCGGAGGACCAGATCG | CTGAGACTCCGGGAGCAG |
| BLE1q | TCTACAGGGCTAAGCAGGAC | CATGCCCATGACCATGTGAG |
| XTR1q | TCAGCATGCACATCAAGCTC | GTTCGTCTGCAGGATGTACG |
| BRD1q | GAGGTGGCTGGAGAAGAACAT | ATTTCTACGGTGCCTACTTCCT |
| CESA6q | GAGCTTACTTGGTGGAAG | AATAAACAATTGGCATCTTC |
| IRX10Lq | GCAGGCTCATCTTATTCC | TGCTGGTATCGTAGAACA |
| GT8q | AGATTGATTCGTTAGGCTTC | AAATGAAACCTTTATCCTATCCA |
| UGA4eq | ATTGTCCTTGGAGGCTAG | AATTCAGCATGGTTGATTG |
| CSLF6q | GGTGTGGTGTATGATTGT | CAGCCTCTCTTTCTCTCA |
| Exp1q | AGTGACGCTTCAGGAACGAT | TAGTCGCACATGATCCGGTA |
| ExpA8q | CTTCTGCCCTCCAAACTACG | GAAGTAGCTGTGCCCGTTC |
| ExpA10q | CTTCACTGATCGGACATCGC | CTTCACTGATCGGACATCGC |
| ExpA17q | GCTGGAATAATCCGGCCATC | GCTGCCAGTACAAGTCTGTG |
| ExpA30q | GCGGATGAGCCACAACTG | AGGAGGTGACCTTGAAGCTG |
| ExpB2q | TCGTCTACACCAACGACTGG | CATGAACGGGTACTGGTTGG |
| ExpB3q | TTCTCGTCGATGACCTCCTG | AGGGTGGTTGACGCATCTTA |
| ExpB5q | CGGAGTCCTCAAGATCCAGT | CGAGGTAGTACGGGTTGGAG |
| ExpB12q | CGATGACAACGACAGTGACC | CGTTGCTGGCAACAAACATC |

**Supplementary Figure**


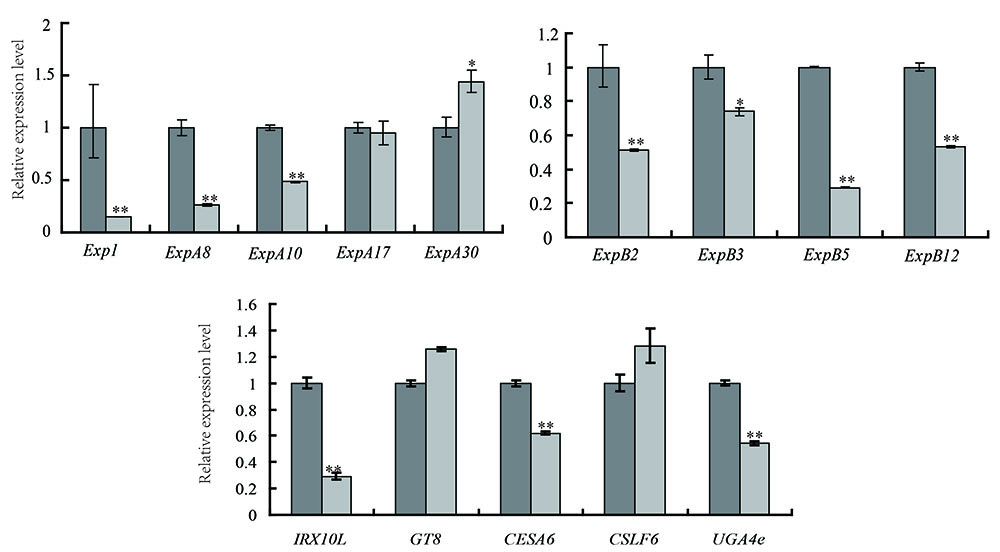


Figure S1 Relative expression levels of cell wall synthesis-related genes(*CESA6*, *IRX10L*, *GT8*, *UGA4e*, and *CSLF6)* and cell expansion-related genes, *Exp1*, *ExpA8*, *ExpA10*, *ExpA17*, *ExpA30*, *ExpB2*, *ExpB3*, *ExpB5*, and *ExpB12*. Double asterisk indicates significant differences between WT and *ds1* at *P*=0.01 by Student’s *t* test,


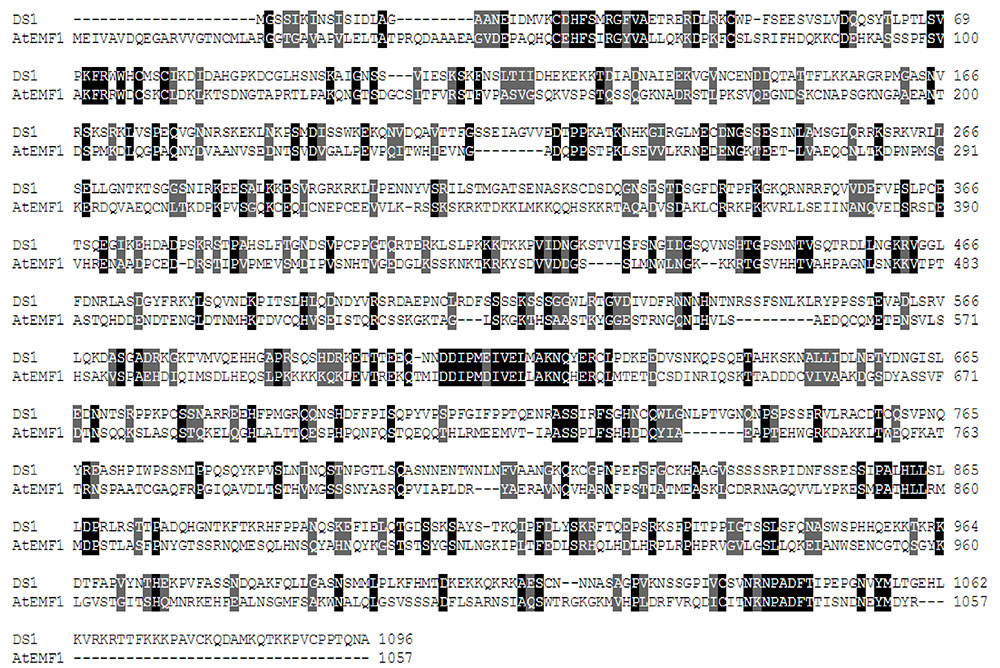


**Figure S2** Alignment amino acid sequences of DS1 and AtEMF1
